# Improved thermostability of creatinase from *Alcaligenes Faecalis* through non-biased phylogenetic consensus-guided mutagenesis

**DOI:** 10.1186/s12934-020-01451-9

**Published:** 2020-10-17

**Authors:** Xue Bai, Daixi Li, Fuqiang Ma, Xi Deng, Manjie Luo, Yan Feng, Guangyu Yang

**Affiliations:** 1 Institute of Biothermal Science and Technology, University of Shanghai for Science and Technology, Shanghai, 200093 People’s Republic of China; 2 CAS Key Lab of Bio-Medical Diagnostics, Suzhou Institute of Biomedical Engineering and Technology, Chinese Academy of Sciences, Suzhou, 215163 Jiangsu People’s Republic of China; 3 State Key Laboratory of Microbial Metabolism, School of Life Sciences and Biotechnology, Shanghai Jiao Tong University, 800 Dongchuan Rd., Shanghai, 200240 People’s Republic of China; 4 Wuhan Hzymes Biotechnology Co., Ltd., Wuhan, 430000 Hubei People’s Republic of China

**Keywords:** Creatinase, Thermostability, Consensus approach, Multiple sequence alignment, Phylogenetic analysis

## Abstract

**Background:**

Enzymatic quantification of creatinine has become an essential method for clinical evaluation of renal function. Although creatinase (CR) is frequently used for this purpose, its poor thermostability severely limits industrial applications. Herein, we report a novel creatinase from *Alcaligenes faecalis* (*af*CR) with higher catalytic activity and lower *K*_M_ value, than currently used creatinases. Furthermore, we developed a non-biased phylogenetic consensus method to improve the thermostability of *af*CR.

**Results:**

We applied a non-biased phylogenetic consensus method to identify 59 candidate consensus residues from 24 creatinase family homologs for screening *af*CR mutants with improved thermostability. Twenty-one amino acids of *af*CR were selected to mutagenesis and 11 of them exhibited improved thermostability compared to the parent enzyme (*af*CR-M0). Combination of single-site mutations in sequential screens resulted in a quadruple mutant D17V/T199S/L6P/T251C (M4-2) which showed ~ 1700-fold enhanced half-life at 57 °C and a 4.2 °C higher T_50_^15^ than that of *af*CR-M0. The mutant retained catalytic activity equivalent to *af*CR-M0, and thus showed strong promise for application in creatinine detection. Structural homology modeling revealed a wide range of potential molecular interactions associated with individual mutations that contributed to improving *af*CR thermostability.

**Conclusions:**

Results of this study clearly demonstrated that the non-biased-phylogenetic consensus design for improvement of thermostability in *af*CR is effective and promising in improving the thermostability of more enzymes.

## Background

**Fig. 1:**
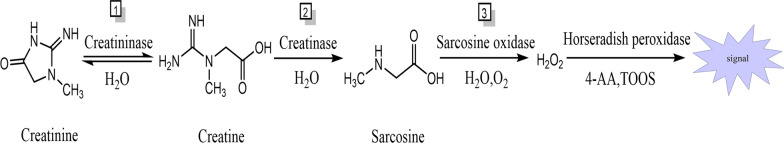
Scheme for enzymatic detection of creatinine. Creatinine is converted by three enzymes in sequential cascade: creatininase, creatinase, and sarcosine oxidase. First, creatininase catalyzes creatinine to creatine; creatinase catalyzes hydrolysis of creatine in the second step; sarcosine oxidase catalyzes sarcosine to detectable hydrogen peroxide (H_2_O_2_) in the third step. Horseradish peroxidase catalyzes H_2_O_2_ to generate a purple color caused by a coupler reagent 4-AA (4-Aminoantipyrine) and a color-generating substance TOOS (*N*-Ethyl-*N*-(2-hydroxy-3-sulfopropyl)-3-methylaniline)

Creatinine is the final product of phosphocreatine metabolism in humans [1], and has been established as a reliable clinical marker for the determination of renal and muscular dysfunction. One of the most commonly used methods for the detection of creatinine is based on an enzymatic cascade [2] that includes creatininase (E.C.3.5.2.10), creatinase (E.C.3.5.3.3), and sarcosine oxidase (E.C.1.5.3.1) (Fig. 1). In this way, creatinine is eventually converted into H_2_O_2_, and thus the concentration of creatinine can be determined by converting H_2_O_2_ into detectable signal by horseradish peroxidase (Fig. 1). In this system, creatinase is the rate-limiting enzyme and several reported creatinases exhibit low catalytic activity and poor thermostability [3–6]. Consequently, genome mining for new homologs of this enzyme, with subsequent protein engineering to improve its catalytic properties and thermostability have recently become the focus of increasing research attention.

Over the years, many different approaches have been used to modify the stability of proteins. Classic protein stability design strategies are usually based on disulfide bonds design [7], optimization of protein surface charges [8], B-factor design [9], Proline effect design [10]. Recently, design tools rely on the energy function or the machine-learning algorithm have been developed to predict changes in protein stability, such as CC/PBSA [11], I-Mutant 2.0 [12], Fold-X [13], MUpro [14], PopMUSIC [15], Rosetta [16]. However, these approaches usually require a crystal structure of target protein and in-depth understanding of the structure–function relationship, which limited their widely applications.

Consensus design for protein engineering is a method for identifying conserved amino acid residues across a set of homologous sequences to find sites that can serve as strong candidates for mutagenesis to improve thermostability [17–19], and which could make accurate predictions independent of structural information. In 1994, Steipe et al. first proposed the consensus concept and successfully applied it to design stable immunoglobulin variable domains [20]. Subsequently, Lehmann et al. created consensus sequences from a set of homologous phytases to achieve an astonishingly ~ 26 °C higher melting temperature than the wild-type phytase [21, 22]. Thus far, this method has also been successfully applied to improve the thermostability of sucrose phosphorylase, β-lactamase, and fibronectin type III (FN3) domain [23–25].

Although often effective, the consensus approach suffers from a major flaw, that is, the overrepresentation of one or a few sub-families of homologous sequences in the sequence space, which results in bias in the final consensus sequence. This bias may obscure conserved residues, thereby limiting access to the potentially high thermostable properties offered by less-characterized families and evolutionary lineages [26]. Consequently, a purely statistical approach of simply replacing all non-consensus residues in conserved positions of a sequence or motif with the consensus residue may fail to result in a more stable protein [17]. In a previous study, Bloom et al. demonstrated that utilizing a likelihood-based method to account for bias in a phylogeny can relatively reduce database bias for homologous sequences [27].

In this work, we cloned and characterized a novel creatinase from *Alcaligenes faecalis* (*af*CR). The enzyme showed relatively high catalytic efficiency and a low *K*_M_ value compared with previous reported CR from *Flavobacterium*, *Pseudomonas*, which make it a promising candidate for the applications in the enzymatic determination of creatinine [28, 29]. However, the thermostability of *af*CR is poor, thus limiting its use with many reagents. To address this issue, we used a non-biased consensus method based on phylogenetic analyses to identify conserved residues to target for improving thermostability of *af*CR. We determined the optimal conditions for wild-type *af*CR activity, and generated combinatorial mutants to increase *af*CR thermostability. In addition, we used structural homology modeling to examine the interactions potentially underlying the improvements conferred by individual mutations, and found a wide range of interactions that increase the thermostability of the creatinase enzyme without reducing its activity. This work modifies our conventional understanding of the effects of some amino acid substitutions on thermostability, and provides a reliable system for similar engineering of other proteins for industrial applications.

## Results and discussion

### Cloning, expression, and characterization of wild-type afCR gene

Creatinase is used as a key enzyme for enzymatic measurement of creatinine which catalyzes the hydrolysis of creatine to sarcosine and urea. It has been found in various kinds of bacteria such as *Flavobacterium*, *Pseudomonas*, *Arthrobacter* and *Bacillus* [6, 28, 30–32]. In this work, we analyzed the creatinase family sequences base on phylogenetic tree (Additional file 1: Fig. S1) and found a creatinase gene from *Alcaligenes faecalis* in the NCBI database. Its amino acid sequence comparison between creatinase from *Pseudomonas, Flavobacterium,* and *Arthrobacter* creatinase showed about 60% homology. We amplified *af*CR gene and cloned it into pANY1 expression vector. The recombinant DNA was expressed in *E. coli* BL21 (DE3), then purified by affinity chromatography and its activity was determined. The results of *af*CR enzymatic characterization showed maximum activity of 14 U/mg (activity was determined by using the 1 mg/ml enzyme at 37 °C) and a lower *K*_M_ value (23.6 mM), providing a new source of creatinase for the enzymatic determination of creatinine.

### Effects of temperature, pH, and metal ions on wild-type afCR activity

**Fig. 2:**
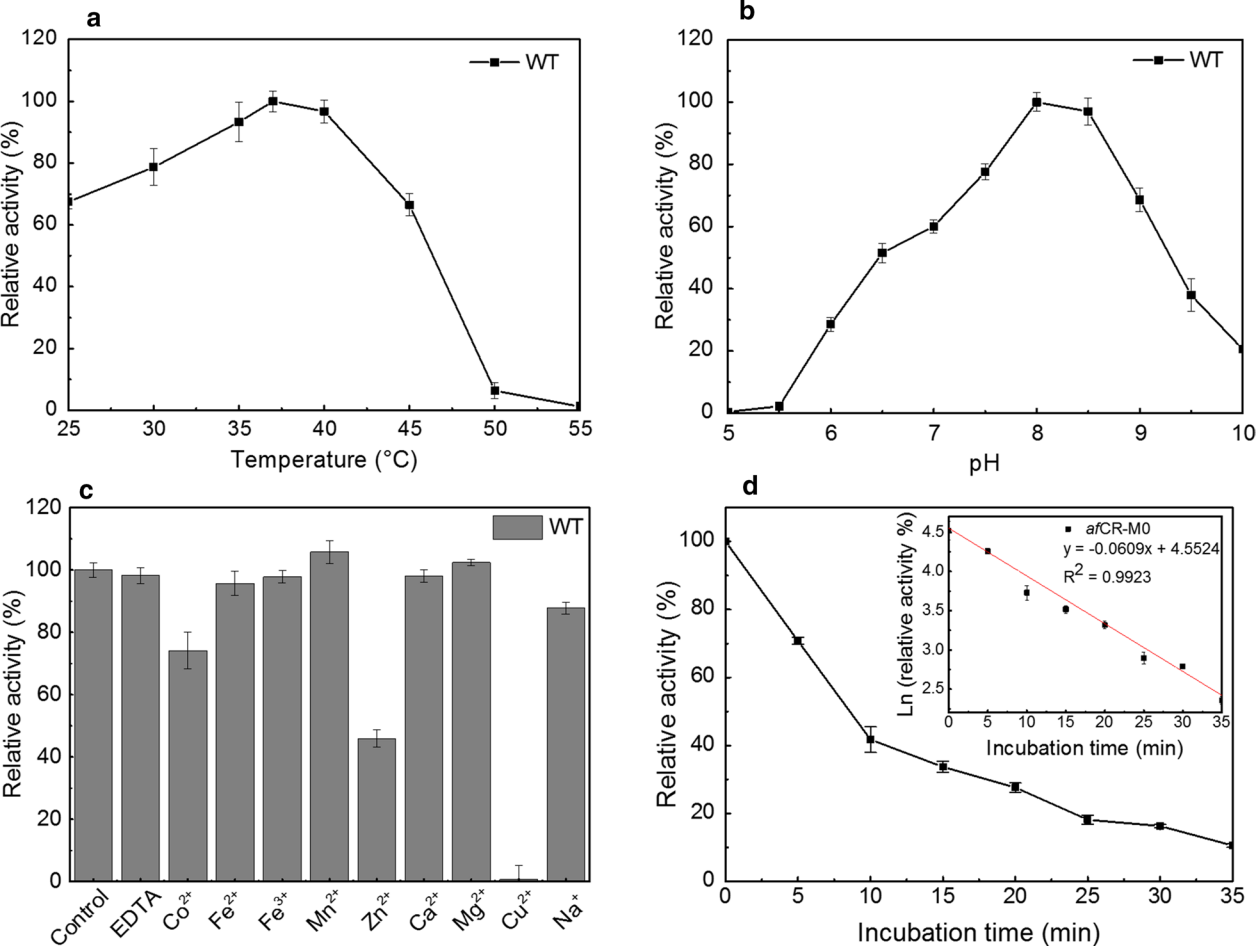
**a** Temperature-dependent activity profile of wild-type *af*CR (WT) determined at pH 8.0 for 60 min. **b** Effect of pH on WT activity determined at 37 °C for 60 min. **c** Effects of several metal ions on WT activity. **d** Thermal inactivation profile of *af*CR-M0 at 55 °C

In order to provide an initial characterization of the optimal temperature for wild-type *af*CR (WT), we first examined its activity across temperatures ranging 25–55 °C. We found that WT activity gradually increased from 30 to 37 °C but was rapidly inactivated at higher temperatures, thus indicating that 37 °C was the optimum temperature for *af*CR reactions (Fig. 2a). Therefore, we examined WT activity over a range of reaction pH values (4.5–10.0) to determine at which values it functioned most efficiently (Fig. 2b). The results revealed a marked change in activity over this range, steadily increasing up to pH 8.0, after which the rate of a*f*CR relative activity declined, with more than 50% of maximum activity observed at a broad pH plateau between 7.5 and 9.0. These results thus demonstrated that pH 8.0 and 37 °C were optimal conditions for *af*CR reactions.

In order to investigate the effects of metal ions on WT *af*CR enzyme stability, the WT was individually incubated with equimolar concentrations of Co^2+^, Fe^2+^, Fe^3+^, Mn^2+^, Zn^2+^, Ca^2+^, Mg^2+^, Cu^2+^, and Na^+^. The WT exhibited differences in sensitivity to the metal ions (Fig. 2c). Specifically, Mn^2+^ and Mg^2+^ ions enhanced WT *af*CR activity. In the presence of EDTA, Fe^3+^, Fe^2+^, Ca^2+^ WT retained > 80% of its initial activity, which has little effect on afCR activity; in contrast, Cu^2+^, Co^2+^, Zn^2+^ and Na^+^ ions inhibited WT activity to varying degrees.

In particular, we have observed that the presence of Cu^2+^ have a significant inhibition effect on the activity of afCR, which is similar to other previous reports on creatinase [2, 33]. It has been reported in previous studies that the metal-ions-induced enzyme inactivation may be due to protein aggregation [34]. However, the aggregation of afCR was not observed in our experiments. We speculated that Cu^2+^ may interact with some key amino acid residues and lead to the loss of catalytic activity of afCR. However, the detailed mechanism of this effect requires further study.

### Non-biased phylogenetic consensus method reveals 21 target consensus residues for mutagenesis

**Fig. 3:**
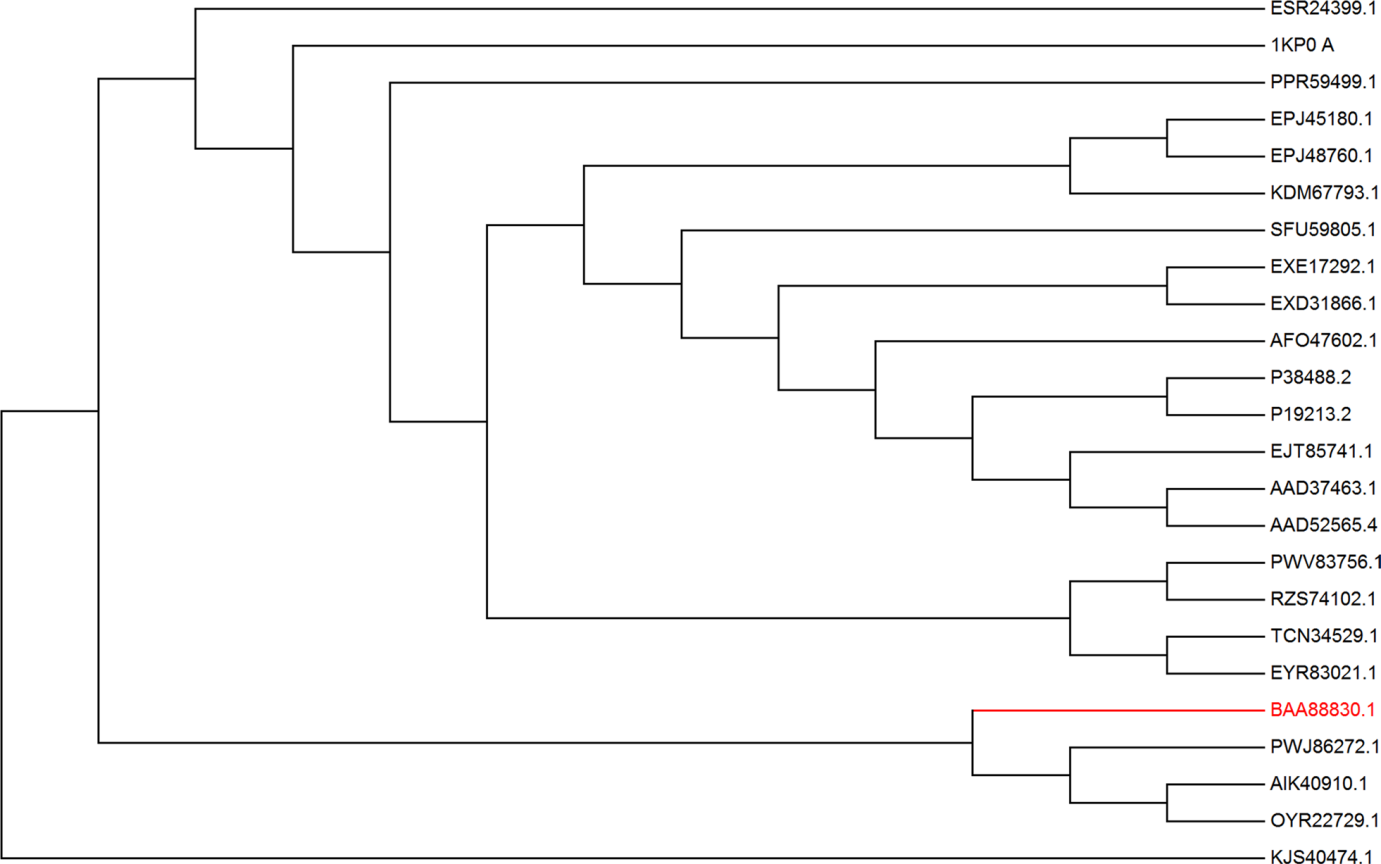
Phylogenetic tree of the 24 creatinase homologs. Neighbor-Joining phylogenetic tree of the 24 homologous creatinase protein sequences (identity > 50%) constructed using MEGA 7.0. The query sequence branch BAA88830.1 from *Alcaligenes faecalis* is indicated in red

In order to employ a non-biased phylogenetic consensus method for identifying specific residues that will most effectively improve thermostability through mutation, we used wild-type *af*CR as a query for a blastP search of proteins in the NCBI database. This search yielded 45 CR sequences with a sequence similarity greater than 50% (Additional file 1: Table S1) compared with wild-type *af*CR. We then aligned these sequences and deleted duplicates, as well as sequences that were too long or too short. A final set of 24 CR homologous sequences were selected for the construction of phylogenetic tree. In order to reduce the branch bias that may be introduced by the overrepresentation family in the database, we developed a non-biased phylogenetic consensus method based on phylogenetic relationships. To this end, we first constructed a neighbor-joining phylogenetic tree and determined the branch lengths for each sequence, representing the evolutionary distance between CR homologous sequences as a ratio of the number of non-identical residue pairs to the minimum length of the sequence (Fig. 3 and Additional file 1: Fig. S4).

Thus, the phylogenetic tree serves as a model for CR divergence caused by evolutionary pressures, with branch lengths used to calculate the weight of each sequence. The use of branch weights thus provided statistical independence among the different protein sequences, enabling the identification of conserved target residues by optimizing the occurrence frequency of amino acid residues (Table 1). We used a proprietary consensus wi.py script to introduce branch weights to calculating the consensus sequence (Additional file 1: Figs. S2, S4). Compared with the wild-type query sequence, 59 of the 404 amino acid positions were selected as candidate consensus residues if they appeared at a given position with > 40% frequency among aligned sequences. This consensus cut-off can be adjusted according to the screening method of consensus mutants and the accuracy of other criteria.

**Table 1:** Consensus design information and experimental characterization results of the single-site mutants

Mutation	Secondary structure^a^	Distance to act. site (Å)	Frequency^b^ (%)	55 °C t_1/2_ (min)	Fold improvement	Relative activity (%)
M0	–	–	–	11.6	1	100
L6P^c^	Loop	12.2	58.97	19	1.64	94.28 ± 9.54
D17V^c^	Loop	11.2	43.04	150	12.9	105.00 ± 5.71
P20T	Loop	11.2	61.00	8.7	0.75	95.24 ± 4.77
V33L	α-Helix	15.6	59.58	5.45	0.47	114.29 ± 11.32
C52N	α-Helix	9.4	91.21	7.85	0.68	110.47 ± 6.67
G58D^c^	β-Turn	11.9	61.00	17	1.47	83.80 ± 10.41
W59F	β-Turn	8	85.88	3	0.26	120.95 ± 6.77
D73T	β-Turn	20.8	68.85	8.1	0.70	106.67 ± 17.41
F108Y^c^	α-Helix	7.8	71.10	16	1.37	105.00 ± 5.71
Y109F^c^	α-Helix	8.3	69.85	13	1.12	122.86 ± 1.91
L162A	β-Turn	13.0	66.42	10.4	0.89	108.57 ± 4.35
T117P^c^	β-Turn	21.0	44.84	12	1.03	100.92 ± 15.57
Q165I^c^	α-Helix	9.8	60.03	20.7	1.78	88.57 ± 9.62
K166A	α-Helix	10.2	51.51	11.3	0.97	103.81 ± 7.56
T199S^c^	α-Helix	10.2	42.81	14	1.2	117.14 ± 8.60
T251C^c^	β-Sheet	6.3	78.10	23	1.98	107.62 ± 2.09
E349V^c^	Loop	14.3	96.20	12	1.03	114.29 ± 13.29
K351E^c^	Loop	15.7	83.23	13.7	1.18	142.86 ± 4.85
V362I	β-Sheet	6.8	48.15	10.9	0.94	99.05 ± 15.23
V340L	Loop	6.2	66.40	3.8	0.33	80.00 ± 15.57
C331S	Loop	6.2	83.72	6	0.52	121.90 ± 1.90

^a^Location of the residue according to the homology structure of the *af*CR

^b^The frequency of the amino residue occurrence as calculated from the sequence alignment of the *af*CR

^c^Thermostable variant

In order to further narrow the pool of candidate residues for mutagenesis, we then considered the impacts on protein structure associated with each position of the 59 residues by applying the following criteria: (1) the substitution should be farther than 6 Å away from the active site to avoid the disruption of catalysis; and (2) we excluded amino acids with side chains that directly formed hydrogen bonds or salt bridges with other residues to avoid decreasing protein structure stability. Based on these screening criteria, we selected 21 mutations for further protein engineering by mutagenesis, including E349V, W59F, C331S, K351E, T251C, F108Y, Y109F, D73T, L162A, V340L, P20T, G58D, Q165I, L6P, V362I, T117P, D17V, T199S, V33L, C52N, and K166A (see Table 1 for residue positions and distance from the active site).

Previously, there are many studies have reported that the distances in the range of 5–10 Å from active sites could be selected to design the mutagenesis library [35–37]. For different enzymes, the optimal distances may depend on their different structure and mechanism, which may not be consistent. Herein, we chose the criteria of > 6 Å based on the structural analysis and substrate docking mode of our creatinase. In fact, our successful design of the enzyme also proved that it is a reasonable consideration.

### Construction and characterization of the afCR mutants

Based on the report of a I304L/F395V double mutant variant of creatinase from *Erwinia* with lower *K*_M_ than its wild type enzyme [38], we first examined the effects of introducing two non-synonymous mutations (I304L/F395V) into WT *af*CR. We then examined the *K*_M_ value for the double mutant (15.3 mM) and found that it was lower than that of the WT afCR (23.6 mM), while retaining similar *K*_cat_ value. Accordingly, the I304L/F395V *af*CR variant (*af*CR-M0) was subsequently used as a parent template to construct more variants. To this end, 21 consensus variants were constructed based on the candidate residues identified above and expressed in *E.coli.* Eleven of the 21 variants showed improved thermostability compared to *af*CR-M0 at 55 °C with 80% or higher activity compared to *af*CR-M0 (Table 1). The best variant D17V (*af*CR-M1) exhibited the highest half-life at 55 °C (150 min), which was 12.9-fold higher than that of *af*CR-M0 (Fig. 2d). The thermostability of the other positive mutants was increased by one to twofold compared with that of *af*CR-M0.

In fact, in previous studies, some researchers have applied consensus approach to design thermostable enzymes, which shown a relatively low design success rate of 20–38% [39–41]. Compared with the other studies that used traditional consensus design method, the non-biased-phylogenetic consensus design method was performed in this work with a higher design success rate of 52%. Analysis of these results also further demonstrated that the non-biased-phylogenetic consensus design is an effective approach and promising in improving the thermostability of more enzymes.

### Combination of beneficial mutations and thermostability of combinatorial variants

In order to further improve the thermostability of *af*CR-M1, we then gradually introduced additional positive mutations (i.e., L6P, G58D, Q165I, F108Y, Y109F, T117P, T199S, T251C, E349V, and K351E) (see Table 2). We found that thermostability improved with subsequent mutations and that these variants retained 80% or higher activity compared to *af*CR-M1. Obviously, during the experiment, the half-life of the mutant at 55 °C increased continuously with the improvement of the thermostability, the half-lives of some mutants became very long. For example, the mutant M2-4 (D17V/T199S) has an half-life ~ 700 min at 55 °C, which made it was difficult to be measured accurately. Therefore, we adjusted the temperature to 57 °C in the subsequent experiment to decrease the difficulty of our experiment.

**Table 2:** Experimental characterization results of the *af*CR multi-site mutants

Enzyme	Mutation	57 °C t_1/2_ (min)	Fold improvement	Relative activity (%)
M0	I304L/F395V	2	1	100
M1	M0 + D17V	40	20	105 ± 7.71
M2-1	M1 + L6P	142	71	104.76 ± 18.10
M2-2	M1 + T251C	71	35.5	118.09 ± 3.25
M2-3	M1 + K351E	101	50.5	161.90 ± 1.90
M2-4	M1 + T199S	210	105	111.43 ± 2.31
M3-1	M2-4+ T251C	599	299.5	112.38 ± 2.86
M3-2	M2-4+ F108Y	482	241	106.67 ± 4.76
M3-3	M2-4+ K351E	859	429.5	157.61 ± 5.30
M3-4	M2-4+ L6P	1258	629	123.81 ± 1.90
M4-1	M3-4+ F108Y	2498	1249	134.28 ± 2.86
M4-2	M3-4+ T251C	3371	1685.5	110.47 ± 1.90

Consequently, among the double mutants, D17V/T199S (*af*CR-M2-4) showed the highest thermostability, with a half-life of 210 min at 57 °C (~ 105-fold higher than that of *af*CR-M0) (Table 2). We then used it as a template to individually introduce other mutations (L6P, T251C, F108Y, Y109F, K351E). All triple mutant variants exhibited significant improvements in thermostability, with the highest increase found in mutant M3-4 (D17V/T199S/L6P), which had a half-life of 1258 min at 57 °C (~ 629-fold higher than *af*CR-M0) (Table 2). We then generated variants of M3-4 by introduction of T251C, F108Y, Y109F, and K351E, respectively. This third round of mutagenesis produced variant M4-2 (D17V/T199S/L6P/T251C), which exhibited the highest stability of all variants up to this point, *i.e.,* a half-life of 3371 min at 57 °C (~ 1685-fold higher than *af*CR-M0). All the above triple and quadruple mutants retained similar catalytic activity to that of *af*CR-M0 in addition to showing improved thermostability. During the combination process, we also discarded some mutation sites that led to decreased activity or failed to significantly improve thermostability, such as D17V/G58D, D17V/Q165I, D17V/T117P, and D17V/E349V.

Notably, we found that some single-site mutants together produced a synergistic effect on thermostability. For example, compared with the single-site mutant D17V, the addition of T199S (D17V/T199S) resulted in another fivefold increase in thermostability, while T199S alone led to a 1.2-fold increase compared to *af*CR-M0 (Table 1). Further combination of D17V/T199S with L6P produced another sixfold increase in thermostability, whereas alone it only provided a 1.64-fold increase compared to *af*CR-M0 (Table 1). Thus, these results demonstrated that these amino acid residues function together resulting in a synergistic improvement to thermostability. However, we also observed that some combinations of single-site mutants resulted in antagonistic effects on the thermostability. For example, compared with the L6P (half-life of 19 min at 55 °C) and G58D (half-life of 17 min at 55 °C) single mutation variants, the L6P/G58D double mutant had reduced thermostability (half-life of 6 min at 55 °C), which was even lower than that of the *af*CR-M0 template. Further exploration of the contributions by each of these residues to overall thermostability will help to clarify the molecular mechanisms underlying the synergistic effects of these mutations, which will provide a useful reference for CR engineering through combinatorial mutagenesis.

### Kinetic and thermodynamic stability of the improved afCR combinatorial variants

**Fig. 4:**
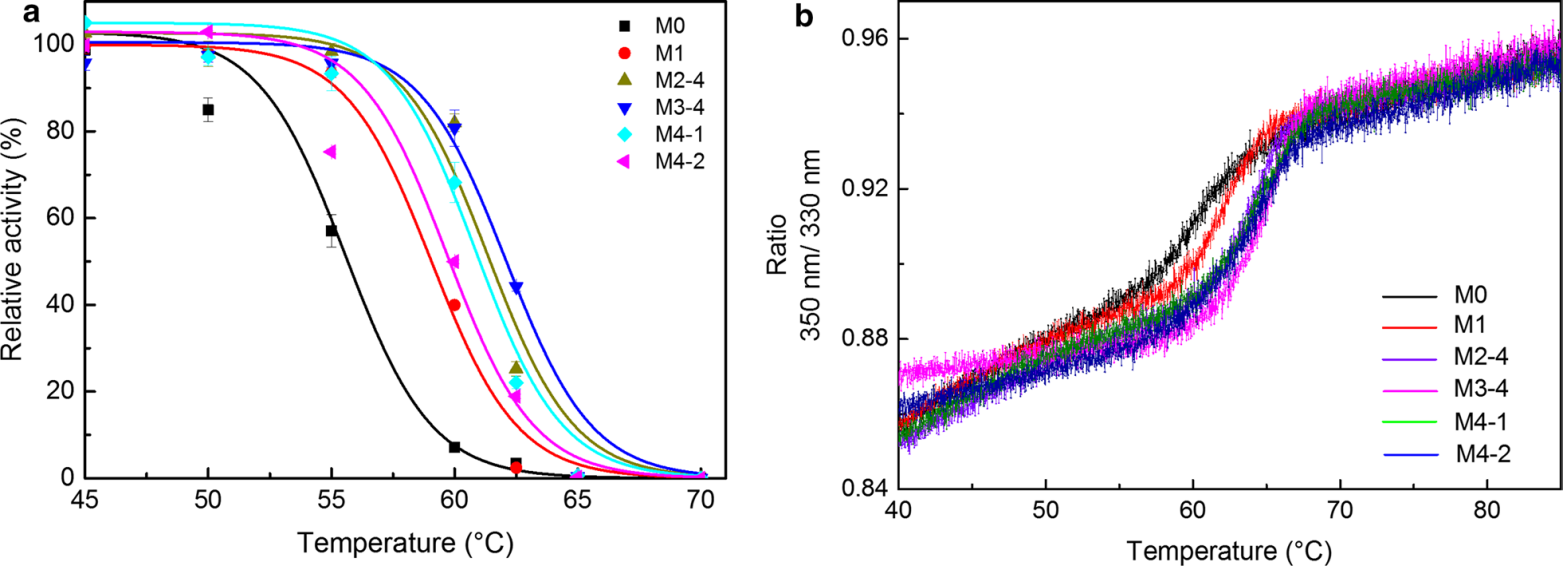
Thermally-induced inactivation and unfolding profiles of *af*CR mutant variants. **a** Thermal inactivation profiles of *af*CR variants. **b** Melting temperature results of the *af*CR variants. Data analysis was performed using Prometheus PR. ThermControl software

To assess the kinetic stability of the variants, activity was measured across a range of temperatures (35–70 °C) to determine the temperatures at which enzyme activity was reduced by 50% after 15 min of incubation (T_50_^15^) (Fig. 4a). We found that the residual activity of M0 was only 7% after incubation at 60 °C for 15 min (Fig. 4a), whereas, M1 retained 40% activity, M2-4 and M3-4 retained 80% activity, M4-1 retained 68% activity, and M4-2 retained 50% activity. These results thus indicated that the T_50_^15^ of these variants was 3.6 to 6.7 °C higher than that of the *af*CR-M0 (Table 3). Notably, although M4-1 exhibited the highest half-life (T_1/2_), it had a lower T_50_^15^ value than that of M3-4. Since T_1/2_ and T_50_^15^ are both primary indicators of protein kinetic stability, this result also demonstrated that the improvements in stability among the combinatorial variants were not due to the simple additive effects of single mutations. However, to determine the contribution of each mutation to the overall effect on thermostability requires detailed exploration of the relationship between protein structure and external reaction conditions in determining protein kinetic stability [42].

**Table 3:** Kinetic and thermodynamic properties of *af*CR mutants

Enzyme	Mutation	T_50_^15^ (°C)	T_m_ (°C)
M0	I304L/F395V	55.5 ± 0.1	59.6 ± 0.40
M1	M0 + D17V	59.06 ± 0.3	62.12 ± 0.02
M2-4	M1 + T199S	61.35 ± 0.2	63.93 ± 0.30
M3-4	M2-4 + L6P	62.21 ± 0.12	64.94 ± 0.20
M4-1	M3-4 + F108Y	61.0 ± 0.15	64.35 ± 0.31
M4-2	M3-4 + T251C	59.7 ± 0.1	64.24 ± 0.23

To further examine how the combined mutations affected thermodynamic stability, the melting temperatures (T_m_) was determined by nanoDSF. The underlying principle of this assay is that the 350/330 nm emission ratio for tryptophan fluorescence of a given protein can indicate the temperature at which the protein unfolds. The T_m_ values for M1, M2-4, M3-4, M4-1, and M4-2 ranged from 2 to 5.3 °C higher than that of *af*CR-M0 (Fig. 4b). Interestingly, unlike the changes in kinetic stability, the melting temperatures of the combined mutation variants increased only slightly over that of the template protein. In previous studies, some researchers has proposed that the unfolding free-energy barrier and the unfolding rate in protein are the key factors of the thermodynamic stability [43, 44]. Therefore, although the denaturation and unfolding process of the enzyme may be related, the results clearly reflected the different process for the enzyme in catalyzed reaction [45], and when measuring enzyme stability, kinetic stability, it should be differentiated from the thermodynamic stability.

### Molecular mechanisms underlying higher thermostability conferred by individual mutations

**Fig. 5:**
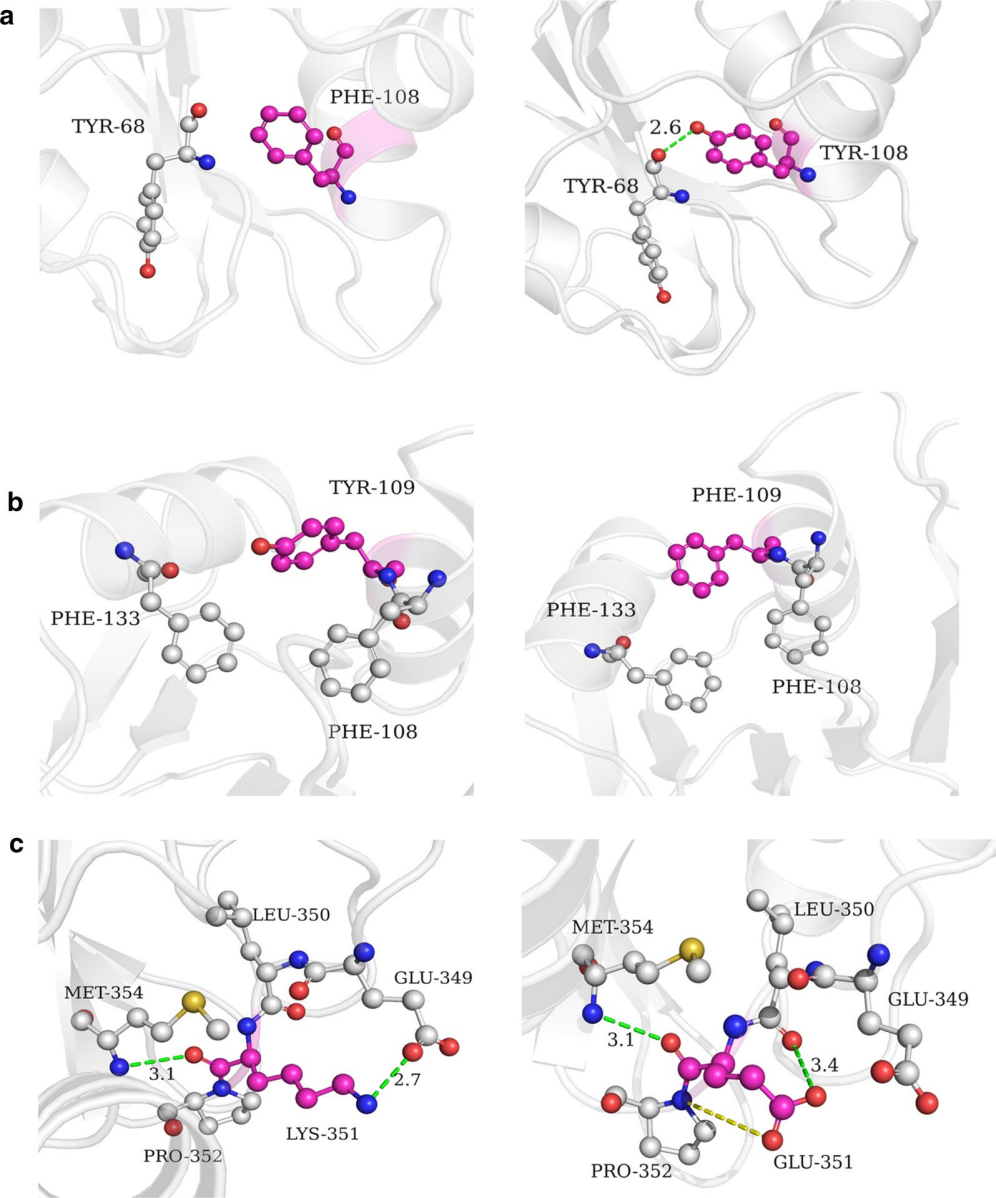
Structural comparisons between the wild type and thermostability-associated residue conversion variants of *af*CR. **a** F108Y, **b** Y109F, **c** K351E wild-type structures (left) compared with mutated residues (right) simulated in a homology model

Given our results of the higher thermostable mutants, we next investigated the mechanistic interactions associated with individual mutations that improved thermostability among the positive mutants using *af*CR structural homology modeling. To this end, we first checked if mutations introduced new interactions (Fig. 5 and Additional file 1: Table S3) and found that the F108Y substitution led to the formation of a new hydrogen bond with the Y68 side chain hydroxyl group (Fig. 5a). Similarly, the Y109F mutation was observed to form new π-π interactions with residues F133 and F108, which subsequently increased the stability of the two helical structures in which the residues are located (Fig. 5b). By contrast, the K351E mutation resulted in the loss of a hydrogen bond between that residue and E349. However, this mutation resulted in new salt bridge interactions and an H-bond formation between P352 and L350 (Fig. 5c). In addition, the T251C substitution introduced a new sulfhydryl group and may create a new disulfide bond with the neighboring C175 (Additional file 1: Fig. S5A).

We then examined changes in hydrophobic and hydrophilic substitutions among the mutants and found that D17V mainly improved hydrophobic packing in the protein interior and facilitated the interaction network between the neighboring residues His12, Asn13, Lys16, Trp90, and Arg91 (Additional file 1: Fig. S5B). Furthermore, we found that Q165I and E349V conversions, mutations similar to D17V, also stabilized the enzyme through increased hydrophobicity, using similar mechanisms as that of D17V. The advantages provided by these mutations apparently contradict conventional mutation theory, in that hydrophobic to hydrophilic amino acid conversion is more likely to improve protein thermostability. For example, the G58D and T199S mutations mainly contributed to higher stability by increasing the hydrophilicity of the protein surface, as well as the inter-helix hydrophobic interactions. In addition, introduction of proline substitutions has been previously shown to be a trend in the stabilization of proteins [46]. The leucine to proline substitution in the L6P mutation (Additional file 1: Fig.S5C) decreased the conformational entropy of local unfolded protein, which resulted in the protein space structure more rigid.

In summary, this analysis showed that individual mutations incurred a wide range of alterations to *af*CR-M0 structure and internal and external interactions, including changes in hydrogen bonds, salt bridge interactions, π-π interactions and disulfide bonds (F108Y, Y109F, K351E, T251C), increased hydrophilic interactions at the protein surface (T199S, G58D), reduction in the conformational entropy of local unfolded proteins (L6P), and improved hydrophobic packing in the protein interior or increased the interaction network (D17V, Q165I, E349V). Moreover, the introduction of the above interactions can improve the thermostability of proteins has been verified in other studies [47–49]. Through analysis of these mutations, we have modified our current understanding of the interaction mechanisms by which different types of amino acid conversions may improve thermostability and thereby provide guidance for engineering higher thermostability in other proteins.

## Conclusions

In this study, we reported a new enzyme with excellent properties and characterized its enzymatic function. Moreover, we demonstrated the enhancement of *af*CR thermostability, guided by an improved non-biased phylogenetic consensus method, through combinatorial mutagenesis of several targeted residues in *af*CR led to significant improvements in thermostability. Specifically, *af*CR mutant M4-2 exhibited a 4.2 °C increase in the T_50_^15^ and ~ 1700-fold enhanced half-life at 57 °C than that of *af*CR-M0, while retaining 80% or higher activity. Consequently, these results showed that this design strategy for improvement of thermostability was effective, to providing a highly thermostable enzyme resource for creatinine detection, in addition can provide guidance for similar improvements in other commercially valuable enzymes.

## Materials and methods

### Experimental operations

#### Media and Reagents

The *af*CR gene (BAA88830.1) was synthesized at GenScript Crop (Nanjing, China) and cloned into a pANY1 expression vector (gift from College of Biosciences and Biotechnology, Shenyang Agricultural University) [50]. Restriction enzymes and T4 ligase were purchased from New England Biolabs (Ipswich, MA). QIAquick™ PCR purification kits were purchased from Qiagen (Hilden, Germany). *E.coli* BL21 (DE3) competent cells were purchased from WEIDI (Shanghai, China). *E. coli* was routinely cultured overnight at 37 °C in 2× YT broth containing Bacto tryptone (1.6%, w/v), Bacto yeast extract (1%, w/v), and sodium chloride (0.5%, w/v) or on 2× YT agar plates with (in both cases) 50 μg/ml kanamycin.

#### Protein expression and purification

Cells expressing recombinant *af*CR were cultivated in 2× YT medium with kanamycin (50 μg/ml) at 37 °C and rotary shaking at 220 rpm. For the expression of *af*CR, the *E. coli* cells were induced by adding isopropyl-β-D-thiogalactopyranoside (IPTG) at a final concentration of 0.5 mM when the OD_600_ value reached 0.6–0.8, then they were further cultured at 20 °C for 16 h. The collected cells were washed and resuspended in binding buffer (25 mM Tris–HCL, pH 8.0 containing 200 mM NaCl and 20 mM imidazole). Resuspended cells were lysed by an High Pressure Cell Disruptor (Union-Biotech, ShangHai, China) followed by centrifugation at 12,000*g* for 20 min to remove cell debris. The supernatant was loaded onto a pre-equilibrated Ni-NTA column (GE, USA), and proteins were eluted with a gradient of imidazole (from 20 to 200 mM). The purity of the collected fractions was analyzed by SDS-PAGE. Fractions containing the pure target protein were combined and desalted by ultrafiltration. The purified proteins were concentrated and stored in phosphate buffer saline (10 mM, pH 7.5) at − 80 °C.

#### Activity measurement

The activity of *af*CR was measured by continuous coupled enzymatic assays, based on the action of sarcosine oxidase and horseradish peroxidase. The enzyme was appropriately diluted to 1 mg/ml with phosphate buffer (10 mM, pH 7.5). Enzyme activity was determined by adding 50 μl enzyme to 950 μl substrate solution. The substrate solution was composed of 500 μM creatine, 0.45 mM 4-AA (4-Aminoantipyrine), 0.5 mM TOOS (N-Ethyl-N-(2-hydroxy-3-sulfopropyl)-3-methylaniline) and phosphate buffered saline (10 mM, pH 7.5), which was incubated at 37 °C. The change in ultraviolet absorption at 555 nm in the enzyme reaction system was monitored using a UV2550 spectrophotometer (Shimadzu). One unit of activity was defined as the amount of enzyme producing 1 μM hydrogen peroxide per minute.

#### Thermostability assay

For determination of enzyme thermostability, the concentration of the purified enzyme was diluted to 1.0 mg/ml in phosphate buffered saline (10 mM, pH 7.5). T_1/2_ values were determined by incubating the purified enzyme at 55 °C or 57 °C for different time periods. Their residual enzymatic activities were assayed at 37 °C as described above. The half-life was calculated by fitting the linear part of the curves: T_1/2_ = − ln (2)/k, where k is the slope of the straight line, and plotting the natural logarithm of the residual relative activity of the enzyme versus heat treatment time.

T_50_^15^ values were determined by incubating the purified enzyme over a range of temperatures from 35 °C to 70 °C for 15 min. After incubation, the sample solution was then cooled immediately in an ice bath and the remaining *af*CR activity was measured as described above. The T_50_^15^ value is the temperature at which enzymatic activity is reduced to 50% after 15 min of heat treatment. The activity measured at 35 °C was considered to be 100%. The data was analyzed by calculating the inflection point of a fit of the residual activity at certain temperatures to a sigmoidal plot (sigmoidal Boltzmann fit using Origin 9.0).

#### Differential scanning fluorimetry

DSF experiments were performed on a nanoDSF device (Prometheus NT.48, NanoTemper Technologies GmbH). All samples were diluted to with phosphate buffered saline (10 mM, pH 7.5) to a final concentration of 1 mg/ml and loaded into high sensitivity capillaries. The protein unfolding process was subjected to a thermal ramp (20–95 °C, 1 °C/min). Data analysis was performed using the Prometheus PR ThermControl software. The Tm-value was determined by fitting the tryptophan 350/330 nm fluorescence emission ratio using a polynomial function in which the maximum slope is indicated by the peak of its first derivative.

#### Measurement of optimal pH and temperature for activity and stability

The optimal pH was determined by measuring *af*CR activity at 37 °C and at pH 4.5 to 10.0 for 60 min. The optimal temperatures of the WT *af*CR were determined at pH 7.0 for 60 min in the temperature range of 25–55 °C.

#### Effects of various metal ions on enzyme activity

The effects of metal ions on enzyme activity were investigated using EDTA, CoCl_2_, FeSO_4_, FeCl_3_, MnSO_2_, ZnCl_2_, CaCl_2_, MgCl_2_, CuSO_4_, and NaN_3_. WT *af*CR was preincubated in phosphate buffer (10 mM, pH 7.5) containing: Co^2+^, Fe^2+^, Fe^3+^, Mn^2+^, Zn^2+^, Ca^2+^, Mg^2+^, Cu^2+^, and Na^+^ at a final concentration of 1 mM for 3 min at 37 °C. After incubation, a residual enzymatic activity was measured as described above.

#### Site-directed mutagenesis

In this work, a two-site variant of *af*CR-M0 (I304L/F395V), with an improved *K*_M_ value compared to wild type *af*CR, was used as the template protein as a basis for subsequent mutations. Site-directed mutagenesis was performed according to the standard QuikChange™ Site-Directed Mutagenesis kit. All mutations were constructed using whole plasmid PCR-based site-directed mutagenesis and the primers containing the mutation site are shown in Table S2. We utilized the pANY1 plasmid harboring *af*CR gene as a template and the correct mutation sites were confirmed by DNA sequencing. For the construction of combinatorial mutants, the most thermostable mutants in each round of construction were used as templates for the next round of site-directed mutagenesis.

### Consensus approach

#### Multiple sequence alignment and Phylogenetic analysis

Using wild-type *af*CR sequence as a query for blastP searches of the NCBI database, we acquired 45 homologous sequences (identity > 50%). These sequences were aligned using the ClustalX 2.1 software package. Duplicate sequences, excessively long and excessively short were excluded from further analysis [51]. Finally, sequences of twenty-four CR homologs were selected for phylogenetic reconstruction and the sequences was displayed using ESPript3.0 (https://espript.ibcp.fr/ESPript/cgi-bin/ESPript.cgi).

Unrooted phylogenies were generated with the phylogenetic module in MEGA7.0 [52] using the Neighbor-Joining method [53]. To construct rooted trees for calculation of sequence weight, we added the outgroup sequence KJS40474.1 to the basal nodes of the unrooted tree (Fig. 3). Here, bias in the natural sequences was balanced by applying a weight to the sequence branch in the tree. The weight of each sequence was calculated according to the branch length in the phylogenetic tree (branch lengths shown in Additional file 1: Fig. S4). The sequence weight was equal to the weighted sum of the average distance of root node branches in the sequence module and the calculation followed Equation (*W*_a_):  which the *W*_a_ was the weight of the target sequence a, X_i_ represents the total branch length of the tree, and the outgroup sequence d was used to add root nodes to the unrooted tree to construct the weighted phylogenetic tree.

#### Consensus sequence

Sequence alignment was performed using ClustalX 2.1 and the Phylogenetic tree was constructed using the same method as above with MEGA 7.0 Software Package [51]. The consensus sequence was calculated by the python script (10.5281/zenodo.3949790).

#### Structural homology modeling for improved variants

The structural homology models of *af*CR were constructed using the SWISS-MODEL [54] package based on the crystal structure of creatinase from *Actinobacillus* (PDB:1KP0, 75% identical to *af*CR). Amino acid residues were mutated using the Mutagenesis module of PyMOL software for subsequent research. All drawings were performed using PyMOL software.

## Supplementary information

**Additional file 1.** Fig. S1. Phylogenetic tree of CR homologous sequences. Fig. S2. AfCR homologous sequences alignment. Fig. S3. Distribution of the stability mutation sites in the CR structural homology model. Fig. S4. Sequence information and branch weight results. Fig. S5. The structural comparison between the wild type and thermostability-associated mutant CR variants. Table S1. CR homologous sequences information. Table S2. Primers of afCR single-site mutants. Table S3. Molecular mechanism of the stabilizing mutations.

## Data Availability

All data needed to evaluate the conclusions in the paper are present in the paper and/or the additional materials. Additional data related to this paper may be requested from the authors.
