# Supplementary material for: Improved thermostability of creatinase from Alcaligenes Faecalis through non-biased phylogenetic consensus-guided mutagenesis
Source: Microb Cell Fact. 2020 Oct 17;19:194. doi: 10.1186/s12934-020-01451-9 (PMC7568399; doi:10.1186/s12934-020-01451-9)
Supplement: Supplementary file 1 — Additional file 1. Fig. S1. Phylogenetic tree of CR homologous sequences. Fig. S2. AfCR homologous sequences alignment. Fig. S3. Distribution of the stability mutation sites in the CR structural homology model. Fig. S4. Sequence information and branch weight results. Fig. S5. The structural comparison between the wild type and thermostability-associated mutant CR variants. Table S1. CR homologous sequences information. Table S2. Primers of afCR single-site mutants. Table S3. Molecular mechanism of the stabilizing mutations. [file 12934_2020_1451_MOESM1_ESM.docx]

**Additional data:**

**
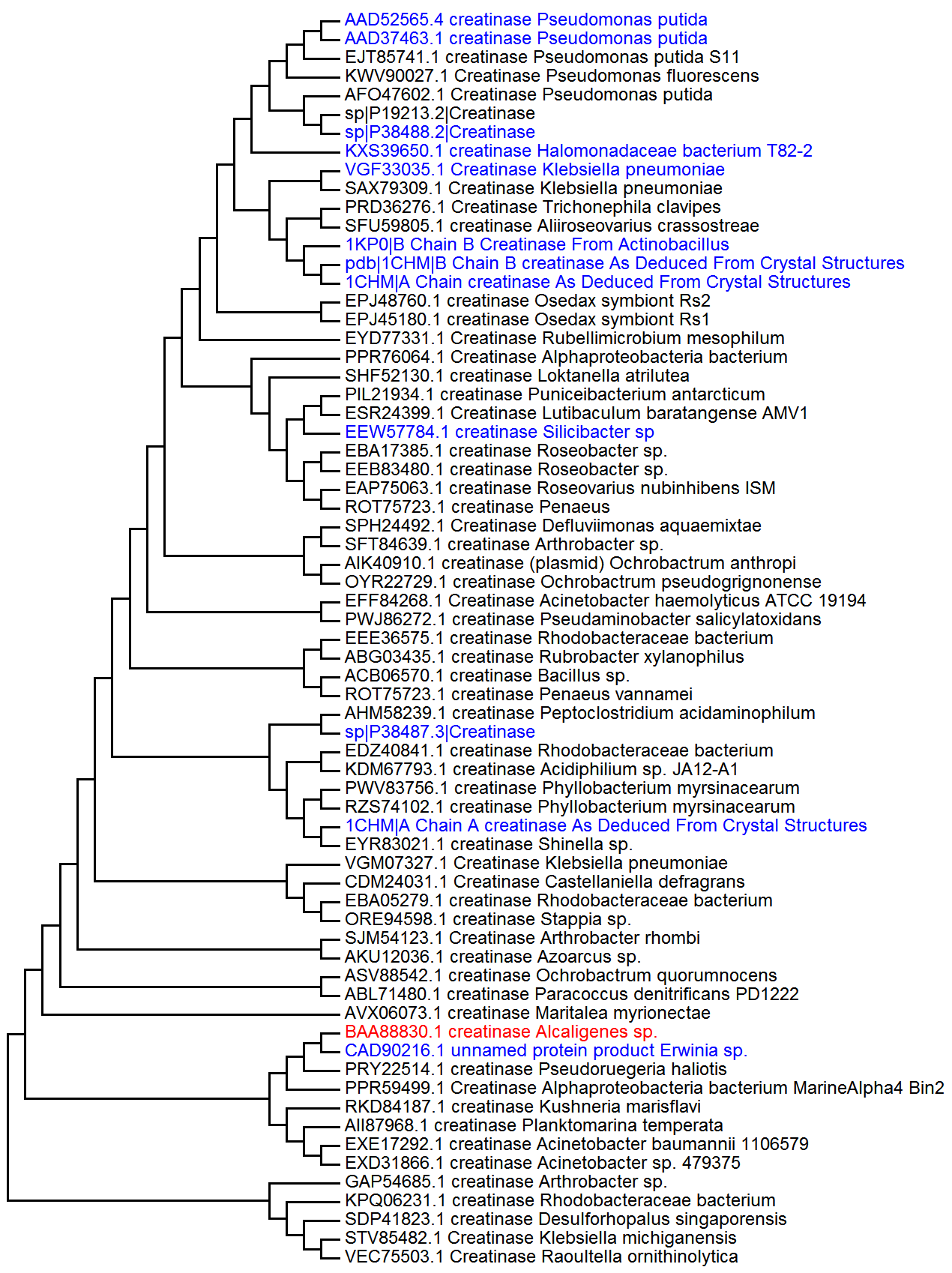
**

**Fig.S1.** Phylogenetic tree of CR homologous sequences. The target *af*CR sequence is shown in red. CR sequences that have been reported in the literature are shown in blue.


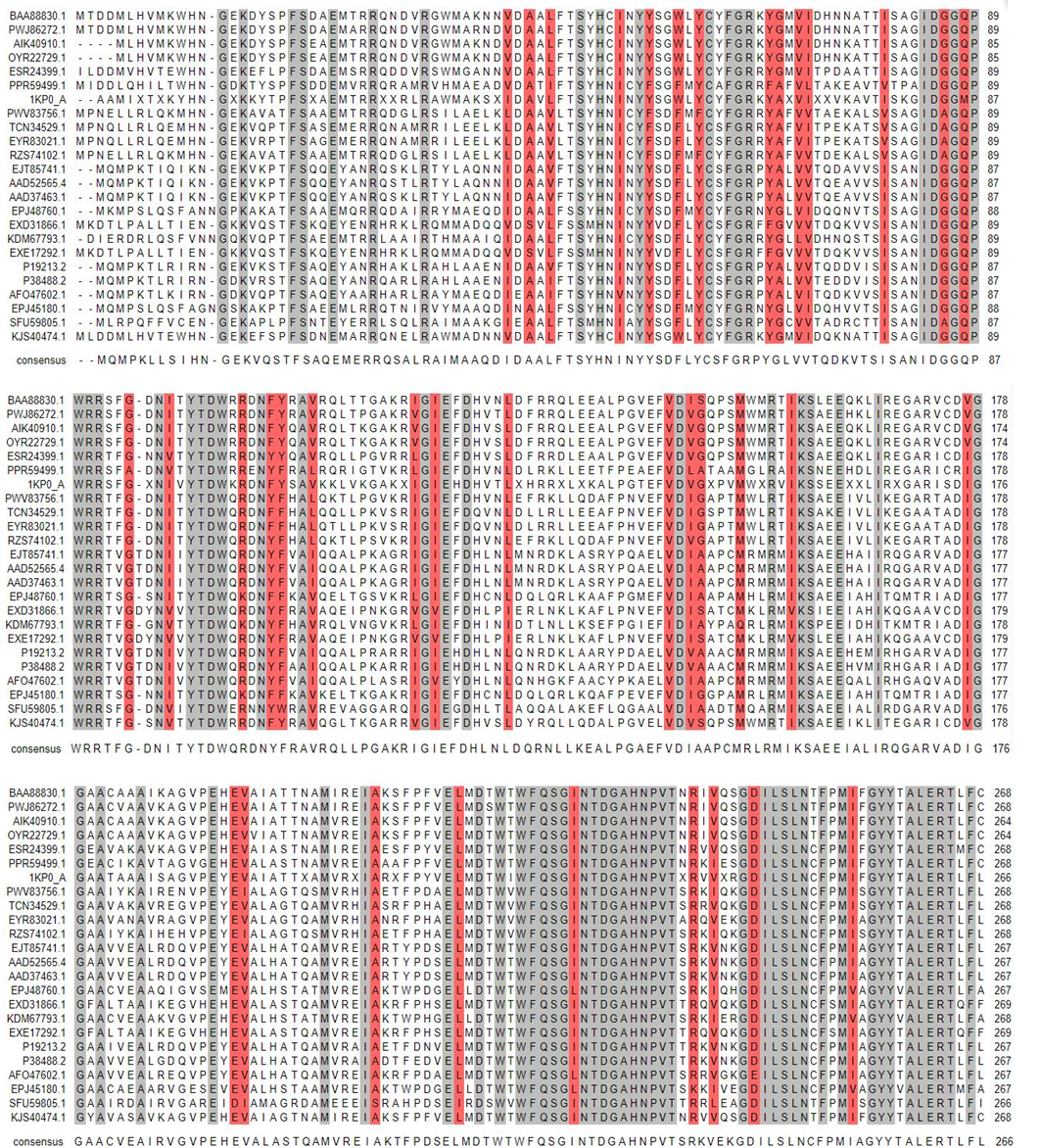

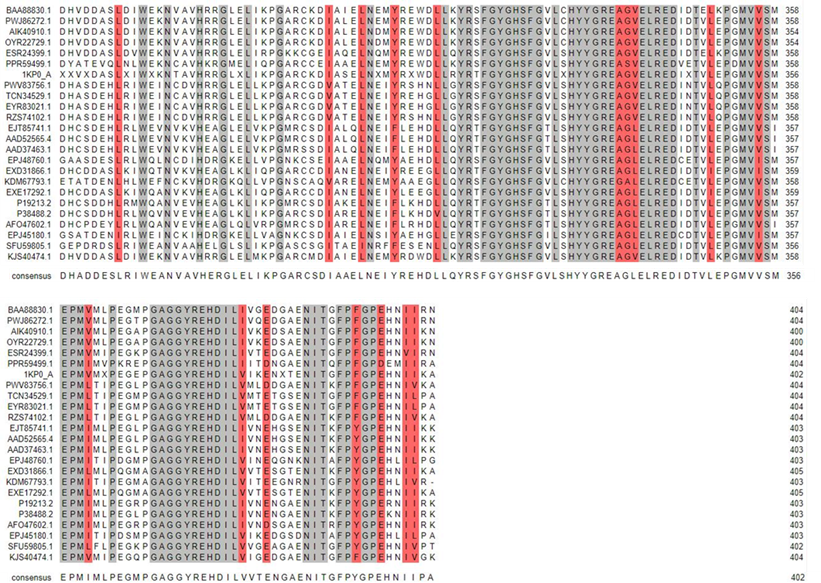


**Fig.S2.** afCR homologous sequences alignment.


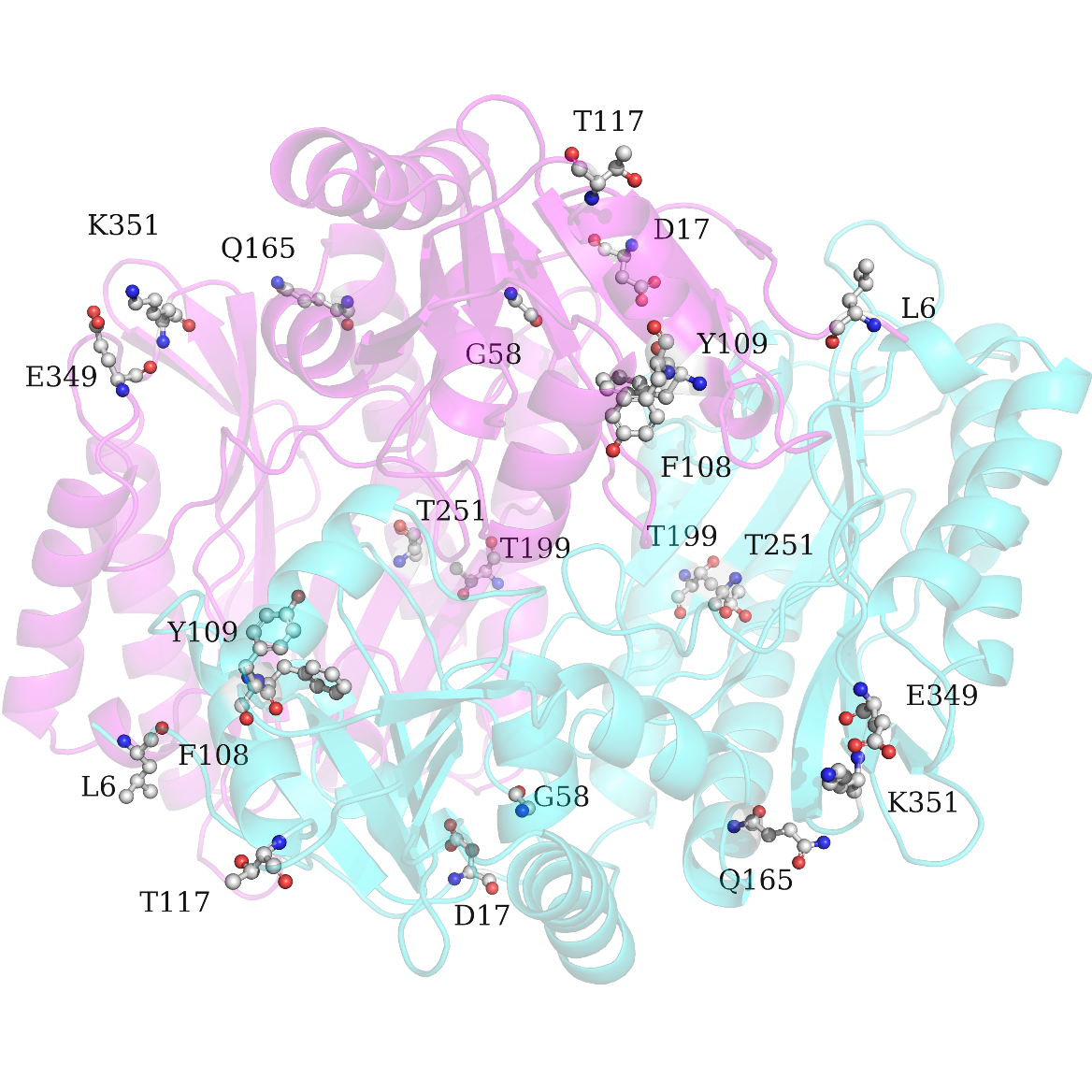


**Fig.S3.** Distribution of the stability mutation sites in the CR structural homology model.

**Fig.S4.** Sequence information and branch weight results.


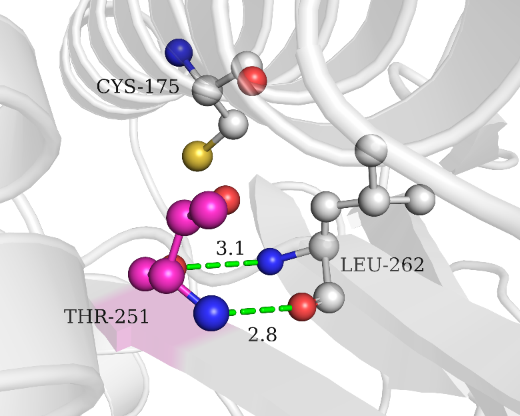

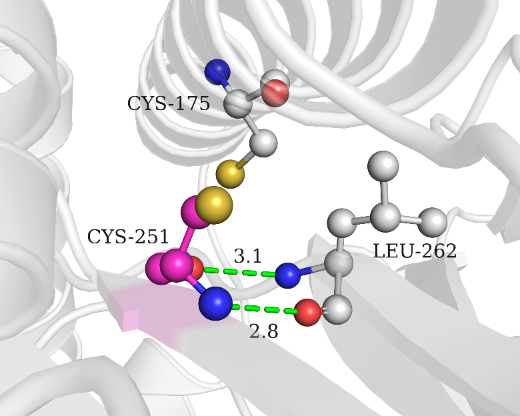


A


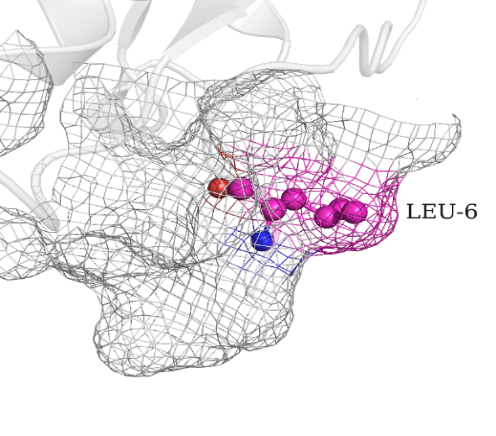

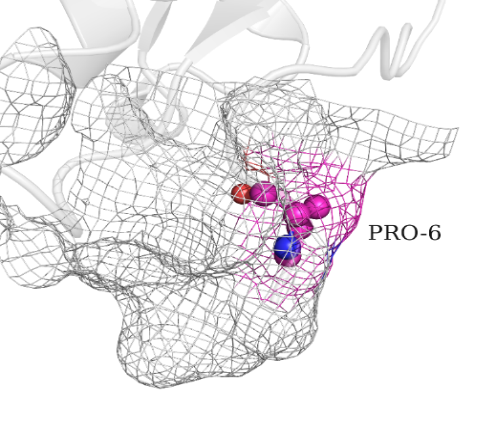


B


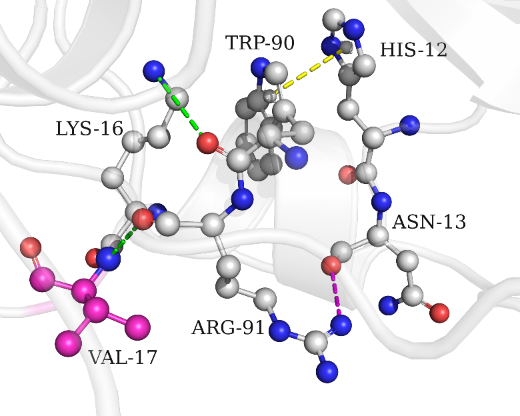

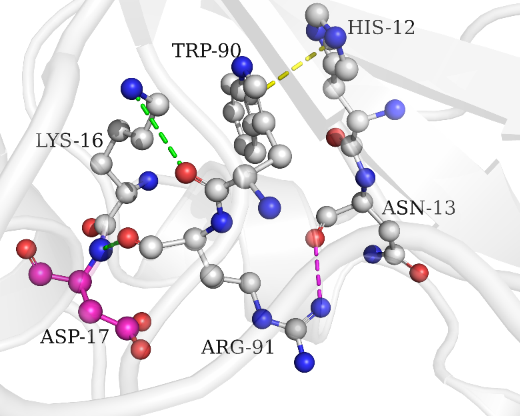


C

**Fig.S5.** The structural comparison between the wild type and thermostability-associated mutant CR variants. (A) T251C, (B) D17V, (C) L6P wild type structures (left) compared mutated variants (right) in structural homology model.

| **Table S1.** CR homologous sequences information. | |
| --- | --- |
| Sequence name | organism |
| BAA88830.1 | creatine amidinohydrolase [Alcaligenes sp.] |
| PWJ86272.1 | creatinase[Pseudaminobacter salicylatoxidans] |
| AIK40910.1 | creatinase [Ochrobactrum anthropi] |
| OYR22729.1 | creatinase [Ochrobactrum pseudogrignonense] |
| ESR24399.1 | creatinase [Lutibaculum baratangense AMV1] |
| ABL71480.1 | creatinase [Paracoccus denitrificans PD1222] |
| PPR59499.1 | Creatinase [Alphaproteobacteria bacterium MarineAlpha4_Bin2] |
| PWV83756.1 | creatinase [Phyllobacterium myrsinacearum] |
| TCN34529.1 | creatinase [Shinella granuli] |
| EYR83021.1 | creatinase [Shinella sp. DD12] |
| RZS74102.1 | creatinase [Phyllobacterium myrsinacearum] |
| RKS52027.1 | creatinase [Paracoccus pantotrophus] |
| SDP41823.1 | creatinase [Desulforhopalus singaporensis] |
| EJT85741.1 | creatinase [Pseudomonas putida S11] |
| AAD52565.4 | creatinase [Pseudomonas putida] |
| RKD84187.1 | creatinase [Kushneria marisflavi] |
| AAD37463.1 | creatinase [Pseudomonas putida] |
| AKU12036.1 | creatinase [Azoarcus sp. CIB] |
| KXS39650.1 | creatinase [Halomonadaceae bacterium T82-2] |
| CDM24031.1 | creatinase [Castellaniella defragrans 65Phen] |
| PPR76064.1 | creatinase [Alphaproteobacteria bacterium MarineAlpha3_Bin4] |
| EPJ48760.1 | creatinase [Osedax symbiont Rs2] |
| EXD31866.1 | creatinase [Acinetobacter sp. 479375] |
| KDM67793.1 | creatinase [Acidiphilium sp. JA12-A1] |
| ABG03435.1 | creatinase [Rubrobacter xylanophilus DSM 9941] |
| EXE17292.1 | creatinase [Acinetobacter baumannii 1106579] |
| XP_015753567.1 | creatinase-like isoform X1 [Acropora digitifera] |
| AFO47602.1 | creatinase [Pseudomonas putida DOT-T1E] |
| EPJ45180.1 | creatinase [Osedax symbiont Rs1] |
| ASV88542.1 | creatinase [Ochrobactrum quorumnocens] |
| ACB06570.1 | creatinase [Bacillus sp. BSD-8] |
| pir\|\|T44250 | creatinase [Arthrobacter sp strain TE1826] |
| AHM58239.1 | creatinase [Peptoclostridium acidaminophilum DSM 3953] |
| XP_015753568.1 | creatinase [Acropora digitifera] |
| SFU59805.1 | creatinase [Aliiroseovarius crassostreae] |
| VGF33035.1 | creatinase [Klebsiella pneumoniae] |
| SJM54123.1 | creatinase [Arthrobacter rhombi] |
| VGM07327.1 | creatinase [Klebsiella pneumoniae] |
| SFT84639.1 | creatinase [Arthrobacter sp. ov118] |
| EBA17385.1 | creatinase [Roseobacter sp. SK209-2-6] |
| EEE36575.1 | creatinase [Rhodobacteraceae bacterium KLH11] |
| 1KP0_A | creatinase [Actinobacillus] |
| P19213.2 | creatinase [Flavobacterium sp. U-188] |
| P38488.2 | creatinase [Pseudomonas putida] |
| P38487.3 | creatinase [Bacillus sp. B-0618] |

| **Table S2.** Primers of afCR single-site mutants. | |
| --- | --- |
| Mutant | Primer |
| L6P | GATGATATGCCGCATGTGATGAAATGGCATAATG  CATCACATGCGGCATATCATCGGTCATGGATCC |
| D17V | GTGAAAAAGTTTACAGTCCGTTTAGTGATGCCGAAATG  GGACTGTAAACTTTTTCACCATTATGCCATTTCATCAC |
| P20T | GATTACAGTACCTTTAGTGATGCCGAAATGACCCGTCG GGCATCACTAAAGGTACTGTAATCTTTTTCACCATTATGCC |
| C52N | CAGCTATCATAATATTAATTACTACAGTGGTTGGCTG  GTAATTAATATTATGATAGCTGGTAAACAGTGCTGC |
| G58D | TACTACAGTGATTGGCTGTATTGTTATTTTGGTCG ATACAGCCAATCACTGTAGTAATTAATACAATGATAGC |
| D73T | GTATGGTTATTACCCATAATAACGCCACCACCATTAG  GCGTTATTATGGGTAATAACCATACCATATTTGCGAC |
| F108Y | CGTGATAATTATTATCGTGCAGTGCGCCAGCTGACCAC CTGCACGATAATAATTATCACGACGCCAATCGGTATAG |
| K166A | GAAGAACAGGCACTGATTCGCGAAGGTGCCCGTGTTTG GCGAATCAGTGCCTGTTCTTCCAGACTCTTAATGGTAC |
| K351E | GATACCGAACTGGAACCGGGTATGGTTGTTAGTATG  CATACCCGGTTCCAGTTCGGTATCAATATCTTCGCG |
| V33L | CAGAATGATCTTCGCGGTTGGATGG  GTCTTACTAGAACGGCCAACCTACC |
| W59F | GTGGTTTCCTGTATTGTTATTTTGGTCGC  CACCAAAGGACATAACAATAAAACCAGCG |
| Y109F | GATAATTTTTTTCGTGCAGTGCGCCAGC  CTATTAAAAAAAGCACGTCACGCGGTCG |
| L162A | TAAGAGTGCTGAAGAACAGAAACTGATTCGCGAAGG  ATTCTCCGACTTCTTGTCTTTGACTAAGCGCTTCC |
| T117P | GCCAGCTGCCTACCGGTGCAAAGAGAATTG  CGGTCGACGGATGGCCACGTTTCTCTTAAC |
| Q165I | GAAGAAATTAAACTGATTCGCGAAGGTGCC  CTTCTTTAATTTGACTAAGCGCTTCCACGG |
| T199S | GCCTCTACCAATGCAATGATTCGTGAAATTG  CGGAGATGGTTACGTTACTAAGCATTTAAC |
| T251C | GAATTGCTTTCCGATGATTTTTGGC  CTTAACGAAAGGCTACTAAAAACCG |
| E349V | GATACCGTTCTGAAACCGGGTATGGTTG  CTATGGCAAGACTTTGGCCCCATACCAAC |
| V362I | GAACCGATGATTATGCTGCCGGAAGGCATGCCT  CTTGGCTACTAATACGACGGCCCTTCCGTACGGA |
| V340L | GAAGCCGGTCTGGAACTGCGCGAAGATATTGATACC  CTTCGGCCAGACCTTGACGCGTTCTATAACTATGG |
| C331S | GTGCTGTCTCATTATTATGGTCGCGAAGCC  CACGACAGAGTAATATACCAGCGCCTTCGG |

| **Table S3.** Molecular mechanism of the stabilizing mutations. | |
| --- | --- |
| Mutant | Putative mutation mechanism |
| F108Y | Hydrogen bond |
| Y109F | π-π interaction |
| K351E | Salt bridge, hydrophobic interactions |
| T251C | Disulfide bond |
| L6P | Conformational entropy, buried hydrophobic interactions |
| T199S | Hydrophobic interactions, surface charge |
| G58D |  |
| D17V | Improved hydrophobic packing in the protein interior, increased the interaction network |
| Q165I |  |
| E349V |  |
